# Theoretical considerations regarding the functional anatomical traits of primary and secondary xylem in dragon tree trunk using the example of *Dracaena draco*

**DOI:** 10.1007/s00425-022-03966-4

**Published:** 2022-07-29

**Authors:** Mirela Tulik, Rafał Wojtan, Joanna Jura-Morawiec

**Affiliations:** 1 Department of Forest Botany, Warsaw University of Life Sciences, Nowoursynowska 159, 02-776 Warsaw, Poland; 2 Department of Dendrometry and Forest Productivity, Warsaw University of Life Sciences, Nowoursynowska 159, 02-776 Warsaw, Poland; 3 Polish Academy of Sciences Botanical Garden - CBDC in Powsin, Prawdziwka 2, 02-973 Warsaw, Poland

**Keywords:** Conduits, Dragon tree, Environment, Hydraulic conductivity, Mechanical strength, Monocot, Vascular bundles, Water transport

## Abstract

**Main conclusion:**

In *Dracaena draco* trunks, the primary and secondary xylem conduits co-function. Both are resistant to embolism; however, secondary conduits are mainly involved in mechanical support.

**Abstract:**

Monocotyledonous dragon trees (*Dracaena* spp., Asparagaceae) possess in their trunks both primary and secondary xylem elements, organized into vascular bundles, that for dozens of years co-function and enable the plant to transport water efficiently as well as provide mechanical support. Here, based on the modified Hagen-Poiseuille’s formula, we examined the functional anatomical xylem traits of the trunk in two young *D. draco* individuals to compare their function in both primary and secondary growth. We provided analyses of the: (i) conduits surface sculpture and their cell walls thickness, (ii) conduit diameter and frequency, (iii) hydraulically weighted diameter, (iv) theoretical hydraulic conductivity, (v) area-weighted mean conduit diameter, as well as (vi) vulnerability index. The conduits in primary growth, located in the central part of the trunk, were loosely arranged, had thinner cell walls, larger mean hydraulically weighted diameter, and significantly larger value of the theoretical hydraulic conductivity than conduits in secondary growth, which form a rigid cylinder near the trunk surface. Based on the vulnerability index, both primary and secondary conduits are resistant to embolism. Taking into account the distribution within a trunk, the secondary growth conduits seems to be mainly involved in mechanical support as they are twisted, form structures similar to sailing ropes and have thick cell walls, and a peripheral localization. *D. draco* has been adapted to an environment with water deficit by distinctive, spatial separation of the xylem elements fulfilling supportive and conductive functions.

## Introduction

The land colonization by the ancestors of modern land plants is one of the most important evolutionary events in Earth history. The terrestrial colonization required a series of innovations in plants’ body structure, including highly specialized tissues with distinct properties that facilitated survival on a new land (Graham et al. 2000; Sperry 2003; Morris et al. 2018). For plants that evolved to larger dimensions and could live for centuries, the prerequisite was the development of a secondary xylem (wood) to transport water as well as provide mechanical support (Pesquet et al. 2011; Lachenbruch and McCulloh 2014). The evolution of water-conducting and supporting xylem cells is believed to have occurred in multiple steps (Friedman and Cook 2000), and it seems to have happened on the principle of something for something (in terms of a trade-off safety *vs*. efficiency of water transport). The first cells to meet the mentioned requirements, reconciling the functions of conduction and strengthening, were the tracheids, typical for most pteridophytes and gymnosperms. These elongated cells usually have tapered ends, a length of 2–4 mm and a lumen of 20–40 µm (Baas et al. 2004). To separate the conductive and reinforcing functions, the xylem vessels and fibers appeared in angiosperms and well-developed gymnosperms, like the Gnetales (Buvat 1989; Karam 2005). Vessels are 25–500 µm wide and complete in length, varying from a few millimeters to several meters (Zimmermann 1983).

Due to the modification of common end walls between two adjacent vessel elements and the increase in their dimensions, the vessels’ conductive function seems to have been perfected as—according to Hagen–Poiseuille formula—efficient water transport depends on the fourth power of the conduit’s diameter (Hacke et al. 2006; Sperry et al. 2008). However, conduits with large dimensions are frequently assumed to be highly vulnerable to embolism (Hacke and Sperry 2001; Hacke et al. 2017; Percolla et al. 2021). This means that widespread embolism in xylem (as in severe water stress) diminishes the plant’s ability to uplift water from the soil to leaves and impairs the rate of carbon fixation by inducing stomatal closure (Cochard 2002; Arango-Velez et al. 2011). Propagation of emboli results in hydraulic failure when plants produce larger vessel diameter in the xylem, but despite a close link between vessel size, hydraulic conductivity as well as xylem embolism spreads, there are studies showing relationship between hydraulic conductivity and other conduit traits, such as conduit wall span and thickness (Hacke et al. 2001), pit anatomical features (Jansen et al. 2009; David-Schwartz et al. 2016; Fernández et al. 2019; Guan et al. 2021) or conduit grouping (xylem network connectivity, Johnson et al. 2020). It is believed that the susceptibility to xylem embolism varies between species (Choat et al. 2012) and correlates with the distribution of species and their sensitivity to damage during drought (Pittermann et al. 2012; Vilagrosa et al. 2012).

The fibers provide mainly mechanical strength to the plant, have more stable cell walls compared with tracheids, and their conductive function has almost been lost (Carlquist 2013; Schuets et al. 2013). Therefore, the evolution of xylem anatomy and its function may be considered as a “trade-off” triangle, where efficiency of water transport interferes with its safety and mechanical strength (Baas et al. 2004; Pratt and Jacobsen 2017; Venturas et al. 2017).

Monocotyledonous dragon trees (*Dracaena* spp., Asparagaceae) are interesting objects for xylem research. They are long-lived and may attain a dozen meters in height in an environment deficient of water resources (Maděra et al. 2020); however, unlike the gymnosperms and non-monocotyledonous angiosperms, their trunks/branches increase in girth due to the activity of the monocot cambium (secondary thickening meristem, Rudall 1991; Jura-Morawiec et al. 2021a). As a result, in a cross-section of dragon tree trunk or branches, the tracheary elements of secondary origin are arranged in vascular bundles and form a rigid cylinder surrounding the primary tissues, which include the primary xylem clustered in vascular bundles (Jura-Morawiec 2017, 2021). Although there have been some previous studies on the secondary tracheary elements of dragon trees (Hubálková et al. 2017; Jura-Morawiec 2017), details of the co-functioning characteristics of the primary and secondary xylem elements are lacking. It is unknown whether the dragon tree's xylem is designed for safe or efficient transport, or both? Therefore, by adopting a xylem "trade-off" triangle and matching the structure–function relationship to environmental requirements, our research was aimed at a morpho-anatomical comparison of the xylem conduits of the primary and secondary origin (i.e., qualitative analyzes) in a *Dracaena draco* trunk, and a quantitative description of their hydraulic variables based on modified Hagen-Poiseuille’s formula, i.e. (i) thickness of cell walls, (ii) conduit diameter and frequency, (iii) hydraulically weighted diameter, (iv) theoretical hydraulic conductivity, (v) area-weighted mean conduit diameter, and (vi) vulnerability index. The above formula describes the capillary flow of a liquid and has been presented in the works of many researchers dealing with water transport of plants (Sperry and Sullivan 1992; Tyree and Zimmermann 2002; Corcuera et al. 2004; Tulik et al. 2014; Apareicido et al. 2015; Yang et al. 2021), therefore, the results obtained by us may constitute a good basis for ecophysiological research on *D. draco*.

## Materials and methods

### Plant materials and slides preparation

**Fig. 1:**
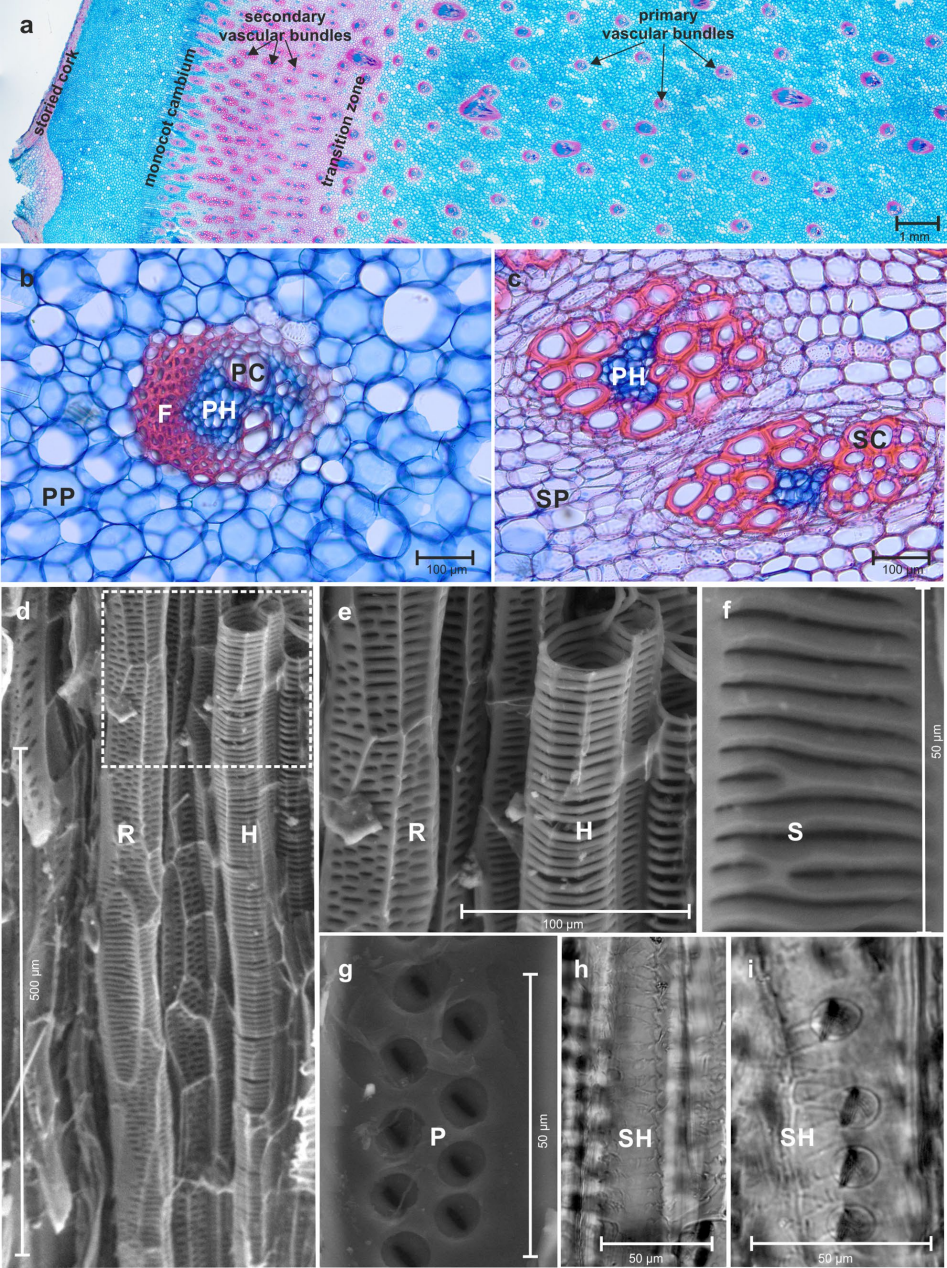
Arrangement and morphology of the xylem conduits in trunk of *D. draco*. **a** Cross-section, 60 µm-thick, stained with toluidine blue and safranin (1:1). Lignified cell walls stained in red. **b** Collateral primary bundle. **c** Amphivasal secondary bundles. **d**–**f** Scanning electron microscope (SEM) views of PC. **g** SEM view of part of the cell wall of SC. **h**–**i** Part of cell walls of SC in a radial section (light microscope). *F* fibers, *H* helical cell wall thickenings, *P* pitted cell wall, *PC* primary conduit, *PH* phloem, *PP* primary ground parenchyma, *R* reticulate cell wall thickenings, *S* scalariform perforation plate, *SC* secondary conduit, *SH* slender helical cell wall thickenings, *SP* secondary ground parenchyma

Two young *D. draco* plants of comparable size (unbranched, approx. 1 m high and with a well-developed zone of the secondary growth in the trunk), were obtained from the commercial nursery (in its natural habitat, this arborescent monocot is classified on the IUCN Red list as an endangered species). The samples containing the protective tissue, cortex, monocot cambium, and both primary and secondary vascular tissues were taken from similar height levels of the trunk in each plant (30 cm from the base), fixed in FAA (ethanol:formalin:glacial acetic acid, 90:5:5, by vol.) and stored in 70% ethanol. Next, part of each sample was cross-sectioned by a core microtome (WSL, Birmensdorf, Switzerland) at a thickness of 60 μm. Radial sections of 15 μm thick were also obtained using a Leica VT 1000S vibrating-blade microtome. The sections were stained with Safranin O and Alcian Blue [1:1, v/v], dehydrated in ethanol series (50–100%), and mounted in Euparal. Macerations of samples were prepared according to Franklin’s protocol (1945). The macerates were stained with 0.01% Safranin O solution which allowed better visualization of the shape and surface of the cell walls. The obtained sections and macerates were then examined with a light microscope OLYMPUS BX 61, equipped with a motorized table, color DP70 digital camera and Cell P, a software for archiving photos and computer image analysis. An image of cross-section (Fig. 1a) was taken with a Telecentric Optical System (2x/0.09). Additionally, small samples, containing primary and secondary growth, were observed with a scanning electron microscope (FEI Quanta 200; Thermo Fisher Scientific, Waltham, MA, USA).

### Biometrical measurements of conduits

Although the transition zone was identified in the cross-section (Fig. 1a), the biometrical parameters were measured in the well-developed zone of primary and secondary growth, i.e., where there were collateral (Fig. 1b; primary growth) and amphivasal (Fig. 1c; secondary growth) bundles. As xylem tracheary elements were analyzed within the context of their conductive function, vessels within the primary growth and tracheids within the secondary growth (Carlquist 2012) were later described as primary conduits (PC) and secondary conduits (SC).

Based on the microphotographs of cross-sections, the diameter of PC and SC (both in tangential and radial direction) and their cell wall thickness were measured from 10 microscopic fields of view (the area of which was 0.142 mm^2^) for every type of growth and for every plant. For further calculations, the tangential and radial diameters of PC and SC were averaged (D, µm). All measurements were done with OLYMPUS Cell P software.

### Hydraulic parameter calculations

The conduit number per unit area (conduit frequency, Renninger et al. 2013) was calculated. The frequency of conduits is expressed as the number of conduits per unit area of primary and secondary growth. First, the number of conduits in a given microscopic field of view (the area of which was 0.142 mm^2^) was counted and then the obtained value was converted to 1 mm^2^ of primary and secondary growth.

Using an estimator defined by Sperry et al. (1994), the hydraulically weighted diameter (*D*_h_) was computed as follows:

1 $$D_{h} \, = \,\Sigma D^{5} /\Sigma D^{4} ,$$

To determine the relationship between conduit diameter and conduit frequency, area-weighted mean conduit diameter (*D*_A_) which correspond to the diameter of an average lumen cross-sectional area, were calculated as follows:

2 $$D_{A} \, = \,(\Sigma D^{2} /No.)^{1/2} ,$$

where *D* is the conduit diameters, No. is the number of conduits measured.

The theoretical hydraulic conductivity (K) was also calculated with the use of the Hagen-Poiseuille law by measuring all *D* within a microscopic field of view as follows:

3 $$K\, = \,(\pi /8\eta x \, (\Sigma D^{4} ) \, x\rho )/A_{s} ,$$

where K is the theoretical hydraulic conductivity (in kg m^−1^ MPa^−1^ s^−1^), η is the viscosity coefficient of water at 20 °C (1.002 × 10–^3^ Pa s at 20 °C), ρ is the density of water at 20 °C (998.2 kg m^−3^ at 20 °C), and *A*_s_ is the cross-sectional area of the microscopic field of view (Zimmermann 1983).

The vulnerability index (VI) was calculated according to the formula given by Carlquist (1977):

4 $${\text{VI}}\, = \,\left( {\text{average conduit diameter}} \right)/\left( {{\text{number of conduits per mm}}^{{2}} } \right).$$

We adopted from Aleman-Sancheschulz et al. (2020) that xylem is more vulnerable to embolism if VI > 1 and is more resistant if VI < 1.

The basic statistics of the conduit’s diameter and the thickness of their cell walls, related to the type of growth (primary and secondary growth), were calculated based on the microscopic measurements (Table 1). The other values were computed based on hydraulic parameters, calculated both for every microscopic field of view and the two analyzed types of growth. To examine the differences between features of PC and SC, the datasets were first tested for normal distribution and the homogeneity of variance. The t test for independent samples was then used to compare the means at α = 0.05. All statistical calculations were done with statistical computing environment R in version 4.1.1 (R Core Team 2021).

**Table 1:** Mean values of biometric and hydraulic variables. Standard errors (SE, *n* = 10) are in parentheses

Analyzed conduit traits	Type of growth		*P* value
	Primary growth	Secondary growth	
Conduit wall thickness (µm)	3.28 (0.07)	8.31 (0.15)	< 0.0001
Conduit diameter (µm)	37.25 (0.73)	28.09 (0.39)	< 0.0001
Conduit frequency (N mm^−2^)	78.1 (9.17)	154.09 (7.23)	< 0.0001
Number of conduits in the microscopic field of view (the area of which was 0.142 mm^2^)	11.1 (1.3)	21.9 (1.03)	< 0.0001
Hydraulically weighted conduit diameter—*D*_h_ (µm)	42.44 (1.03)	32.32 (1.04)	< 0.0001
Area-weighted conduit diameter—*D*_A_ (µm)	38.05 (0.83)	28.77 (0.69)	< 0.0001
Theoretical hydraulic conductivity—*K*_S_ (kg m^−1^ s^−1^ MPs^−1^)	46.59 (5.63)	29.92 (2.35)	< 0.01
Vulnerability index—VI	0.53 (0.05)	0.19 (0.01)	< 0.0001

## Results

### Morpho-anatomical traits of conduits in primary and secondary growth

**Fig. 2:**
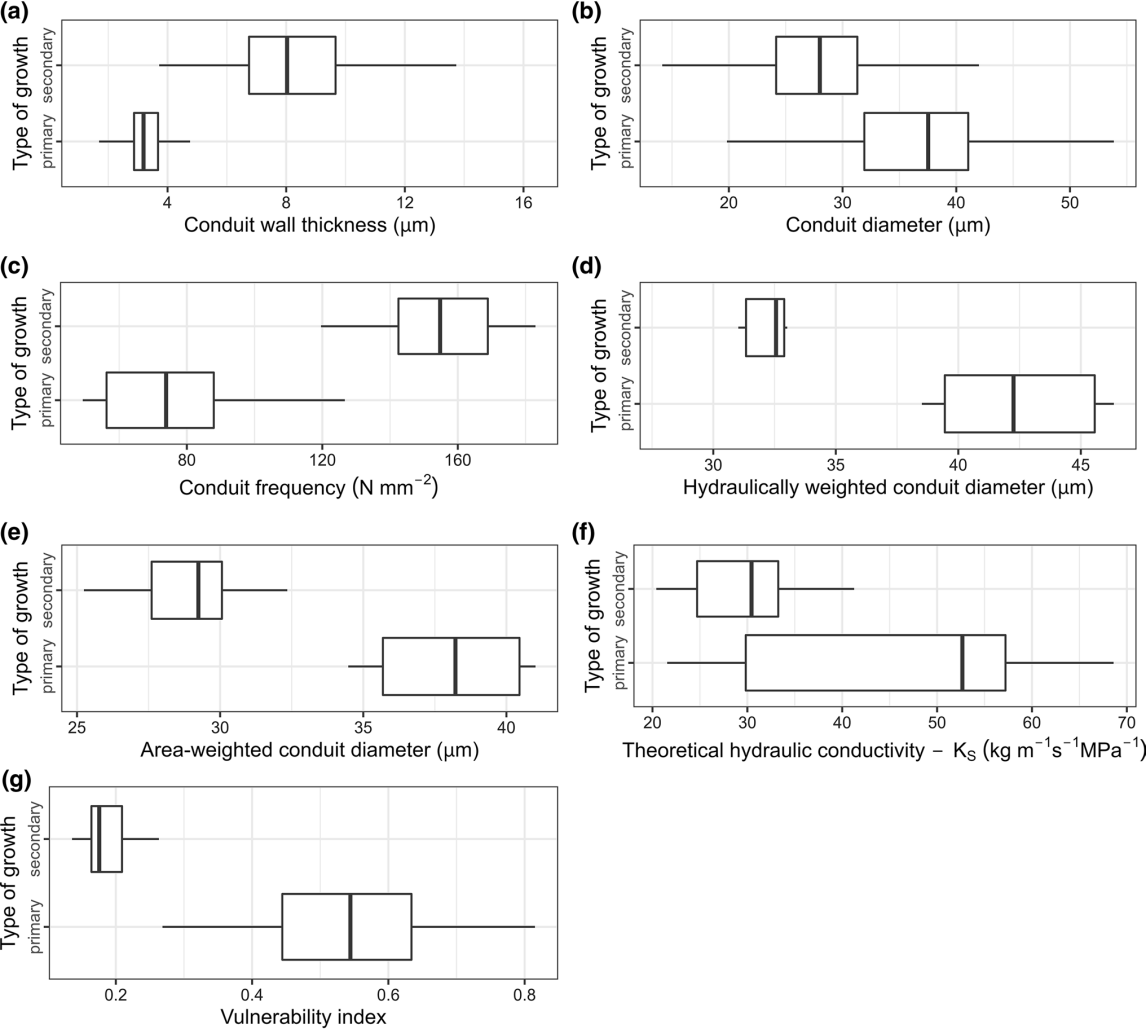
Biometric and hydraulic parameters associated with the primary and secondary growth in *D. draco* trunk. **a** The median values for conduit wall thickness, **b** conduit diameter, **c** conduit frequency, **d** hydraulically weighted conduit diameter, **e** area-weighted conduit diameter, **f** theoretical hydraulic conductivity, **g** vulnerability index. Box plots show median, quartiles and 1.5 Inter-Quartile Range—IQR (or range if less than 1.5 IQR)

The mean cell wall thickness of PC was smaller than that of SC (Table 1, Fig. 2a). We observed many types of sculpturing of their secondary walls. The PC had helical thickenings (double helical with the same curl) (Fig. 1d, e), although some of them appeared to bear grooves rather than helical thickenings. Reticulated and pitted ornamentations were also noted (Fig. 1d, e). Pitting was alternate and outer pit apertures were slit-like. In addition, the combination of more than one type of thickening in the same PC was also found (not shown). In the area of the wall bearing perforation, i.e., a perforation plate, there were several openings arranged in a different manner. This multiple perforation has been classified as scalariform (Fig. 1f) and reticulate perforation plates (perforation plate form a net like patter, from the Latin *rete*, net).

SC were twisted and formed structures similar to sailing ropes. These cells also developed a specifically ornamented secondary wall to provide function (Fig. 1g–i). We recognized the slender helical thickening (double helical with the opposite curl) (Fig. 1h, i) and pitted wall (Fig. 1g). Pits were usually arranged in one, two or three rows (Fig. 1g, i). Their outer apertures were slit-like, similar to those observed in primary xylem conduits. The mean diameter of PC was greater than that of the SC and amounted to 37.25 µm (Table 1, Fig. 2b).

### Hydraulic variables of conduits in primary and secondary growth

Primary xylem had less conduits than secondary xylem: we calculated 78 conduits per 1 mm^2^ in primary growth and, 154 conduits per 1 mm^2^ in secondary growth (Table 1, Fig. 2c). Conversely, PC proved to have a significantly larger mean hydraulically weighted diameter compared with SC: 42.44 µm for PC and 32.32 µm for SC (Table 1, Fig. 2d). Another hydraulic variable analyzed by us was the area-weighted mean conduit diameter, which, for PC, was lower than for SC (Table 1, Fig. 2e). The theoretical hydraulic conductivity (Ks) as a derivative of hydraulically weighted diameter varied between 21.55 kg m^−1^ s^−1^ MPa^−1^ and 68.60 kg m^−1^ s^−1^ MPa^−1^, with an average of 46.59 kg m^−1^ s^−1^ MPa^−1^ for primary growth. In secondary growth, average Ks reached significantly smaller value (29.92 kg m^−1^ s^−1^ MPa^−1^) compared with primary growth (Table 1, Fig. 2f). Based on the vulnerability index (VI), we assumed that PC and SC are resistant to embolism as their VI was less than 1, but a higher value was calculated for PC (Table 1, Fig. 2g). For all analyzed traits, the differences between the values observed for the primary and secondary growth were important and statistically significant (*P* values not greater than 0.01).

## Discussion

Unlike palms or pandans with unitary construction and a fixed body plan (Tomlinson 2006; Tomlinson and Huggett 2012), *D. draco* possesses the ability to produce vascular secondary bundles within a matrix of secondary ground parenchyma cells with lignified walls (Jura-Morawiec et al. 2015). Therefore, in our work, we paid attention to the qualitative and quantitative structure–function relationship of PC and SC in the *D. draco* trunk. Primary thickening meristem produces primary vascular bundles with conduit diameter not larger than 40 µm. They have distinct types of cell wall ornamentations, among others, compound perforation plates. Their vulnerability index of less than 1 indicates that they are well suited for the safe and efficient transport of water assuming the relationship that the larger the diameter of the conducting element, the higher the susceptibility to embolism (Carlquist 1977; Sperry et al. 2003; Baas et al. 2004). However, the question arises: how long are conduits in primary growth functional for the transport of water? Based on our experience in studies of *D. draco*, supported by many anatomical analyses of samples from dragon tree trunks of different age, we suspect that even in older dragon trees the central part of trunk, which is of the primary origin, remains hydraulically functional.

Xylem embolism may develop as a consequence of drought stress reducing hydraulic conductivity (Vilagrosa et al. 2012). *D. draco* functions in a water-limited environment. If, however, there is a risk of embolism caused by drought stress, refilling the embolized primary growth conduits seems easy due to their proximity to the living cells of the ground parenchyma and phloem tissues (Zwieniecki and Holbrook 1989; Zwieniecki et al. 2004). Moreover, the central position of the primary growth conduits is also advantageous, protecting them from bending stresses that could stop the transport of water (Niklas 1995). The primary ground parenchyma cells, having cellulose walls, play not only a protective function against embolism but can support the central part of *Dracaena* trunk hydrostatically. The transition from hydrostatic support to cell wall support in *Dracaena* is achieved by the formation of the thick-walled, lignified, located peripherally SC that are more densely distributed than the PC, similar to the vessels of the frond, trunk, and root of palm trees (Renninger et al. 2013). Their axial twist, resembling a braid (Jura-Morawiec 2017, 2021) or lines in ropes or climbers looking for support, and small pits number along the walls favor their mechanical function, which may be amplified by secondary parenchyma cells with lignified cell walls. This pattern of xylem design with conduits embedded in supporting cells (especially fibers) or adjacent to ray parenchyma or “contact cell” is common for dicots (Carlquist 1988). The small diameter of SC, not susceptible to embolism, can be involved in the hydraulic function under low water availability. Thus, the costs of investment of *Dracaena* in secondary growth create an interesting structural and functional adjustment for the mechanical strength and safe transport of water.

Referring to the conclusions of Carlquist's (2012) study, that monocot cambium is unable to produce vessels, we conclude that tracheids representing xylem SC are formed mainly for mechanical strength. Nadezhdina et al. (2015) reported higher sap flow in the inner part of *D. cinnabari* seedlings. A similar division of function was also reported in palms; the xylem of inner vascular bundles conducts much more water than xylem of outer vascular bundles (Sperling et al. 2012).

The modular organism of *D. draco* to compensate the hydraulic limitations imposed by its increased height bears the costs related to, among others, the production of SC. The cost–benefit margin gives the species a chance for survival under water-deficit environmental conditions. Moreover, Jura-Morawiec and Marcinkiewicz (2020) and Jura-Morawiec et al. (2021b) showed that for a long-term drought, *D. draco* has developed traits and mechanisms that are visible at every level of the organization of its body.

The xylem of woody plants transports water, provides mechanical support, and stores carbohydrates. These mentioned functions are independent, giving rise to trade-offs in function (Baas et al. 2004). Since many features can contribute to the trade-offs of safety (embolism resistance) against efficiency (water transport capacity), it seems that in the *D. draco* this compromise can potentially be modified by the parenchyma xylem fraction (Tyree and Zimmermann 2002). While we do not study the storage of carbohydrates in the ground parenchyma cells of primary origin, we predict the involvement of these cells in storage and promoting plant resistance to limited soil moisture. Jupa et al. (2017) have suggested a high concentration of osmotically active, soluble, non-structural carbohydrates in the ground tissue cells of primary origin in the *D. marginata* stem.

We conclude that the structural organization of the trunk xylem in *D. draco* puts this tree-like monocot between arborescent palms without secondary growth and forest trees with woody trunks. The observed trend in conduit diameter reflects environmental constraints, eliminating dysfunctions and ensuring efficient, safe water transport and mechanical resistance. It should be noted, however, that our knowledge of the relationship between the variability of embolism resistance and the efficiency of water transport among plants is inconclusive as some studies have found a trade-off of varying degrees of significance, and others have not shown any significant relationship (Wagner et al. 1998; Maherali et al. 2004; Fichot et al. 2010; de Guzman et al. 2017). Gleason et al. (2016) conducting studies throughout a wide range of species covering 335 angiosperms and 89 gymnosperms suggest that the correlation safety vs. efficiency is weak, and that although there are no species possessing both safe and efficient hydraulic systems, numerous species with low efficiency and low safety are simply a deviation from this principle and supports the idea of the safety/efficiency dilemma. Moreover, our data regarding the quantitative description of the PC and SC in *D. draco* trunk refer to two young plants, therefore, definitely more measurements should be done on samples of dragon trees growing in situ to establish how broadly our conclusions apply.

### *Author contribution statement*

MT and JJ-M designed the research and developed the methodology, MT, JJ-M and RW conducted investigations and formal analysis, MT, JJ-M wrote an original draft with contribution of RW. All authors read and approved the final version of the manuscript.

## Data Availability

All data generated or analyzed during this study are included in this article and are available from the corresponding author upon reasonable request.

## Abbreviations

PC: Primary conduits
SC: Secondary conduits
